# Impact of the introduction of percutaneous edge-to-edge mitral valve reconstruction on clinical practice in Germany compared to surgical valve repair

**DOI:** 10.1007/s00392-020-01675-0

**Published:** 2020-05-27

**Authors:** Lutz Frankenstein, Klaus Kaier, Hugo A. Katus, Christoph Bode, Tobias Wengenmayer, Constantin von zur Mühlen, Raffi Bekeredjian, Tobias Täger, Manfred Zehender, Hanna Fröhlich, Peter Stachon

**Affiliations:** 1 Department of Cardiology, Angiology, Pulmonology, University of Heidelberg, Im Neuenheimer Feld 410, 69120 Heidelberg, Germany; 2 Department of Cardiology and Angiology I, Heart Center Freiburg University, Freiburg, Germany; 3 Center for Medical Biometry and Medical Informatics, Faculty of Medicine, University of Freiburg, Freiburg, Germany; 4 Department of Cardiology, Robert-Bosch-Hospital, Stuttgart, Germany

**Keywords:** MitraClip, Percutaneous mitral valve repair, Surgical valve repair

## Abstract

**Background:**

The introduction of percutaneous mitral valve (MV) repair had an effect on clinical practice in comparison with surgical MV repair. Complete nationwide data are useful in examining how the introduction of a new technique influences clinical practice.

**Methods:**

We analyzed procedural numbers, patient characteristics, and in-hospital outcomes for all percutaneous edge-to-edge and surgical MV reconstruction procedures performed in Germany between 2009 and 2015.

**Results:**

12,664 percutaneous edge-to-edge and 22,825 surgical MV reconstructions were recorded. Numbers increased steadily, albeit more rapidly in the percutaneous edge-to-edge group (108–4079 vs. 2923–3603 with surgical MV reconstruction). Patients with percutaneous edge-to-edge MV reconstruction were older (75.6 ± 8.8 vs 61.6 ± 13.4 years, *P* < 0.001) and at higher operative risk (estimated logistic EuroSCORE 13.2% vs. 4.7%, *P* < 0.001) compared to those undergoing surgery. However, in-hospital mortality did not differ (2.9% vs. 2.8%; *P* = 0.395). This was also true for the subset of 2103 patients at intermediate operative risk as defined by a logistic EuroSCORE ≥ 4% and ≤ 9%. Of note, complication rates (except acute kidney injury) were more favorable in patients undergoing percutaneous edge-to-edge reconstruction.

**Conclusions:**

Percutaneous edge-to-edge MV reconstruction has markedly changed clinical practice of MR therapy in Germany. Annual overall procedural numbers more than doubled, with a massive increase in percutaneous edge-to-edge procedures. Our data demonstrate its use mainly in high-risk patients and prove the favorable safety profile of this novel technique, with low in-hospital mortality and complication rates.

**Electronic supplementary material:**

The online version of this article (10.1007/s00392-020-01675-0) contains supplementary material, which is available to authorized users.

## Introduction

Mitral valve regurgitation (MR) is the second most common valvular disorder in the elderly, with more than 10% of people aged ≥ 75 years suffering from severe symptomatic MR [1, 2]. Depending on underlying etiology, presence of symptoms, age, and left ventricular function, the prognosis of medically managed MR remains limited [3–7]. Since its introduction in 1957 [8], mitral valve (MV) surgery has been the sole treatment option. Studies have shown that MV surgery significantly reduces cardiac events in patients with primary MR, with benefit largely driven by MV repair [3, 4]. However, approximately 50% of patients with severe MR are denied surgery due to high operative risk and unsatisfactory long-term survival, indicating an unmet need for less invasive treatment strategies especially in older patients and in those with secondary MR [7].

Since 2008, percutaneous edge-to-edge leaflet repair of the MV using the so-called MitraClip system (Abbott Vascular, Santa Clara, California, USA) has emerged as a new treatment option for patients with severe MR. Following positive results from small randomized trials [9, 10], current European guidelines recommend to consider percutaneous edge-to-edge procedures in patients with symptomatic primary severe MR who are judged at high operative risk and in patients with severe secondary MR who remain symptomatic in spite of optimal HF therapy [6]. Recently, the randomized, controlled COAPT trial confirmed a clear mortality benefit against medical therapy alone [11].

The introduction of transcatheter MV interventions has led to questions about the effect of this technique on clinical practice and surgical MV therapy, which is still considered the standard of care. The aim of our analysis was to evaluate how the treatment of patients for MR has evolved in Germany since the introduction of percutaneous edge-to-edge leaflet repair, using real-world data from an unselected, population-based cohort.

## Methods

### Data source and processing

Since the introduction of the German ‘diagnosis- and procedure-related remuneration system’ (DRG-system) in 2004, all hospitals are obligated to transfer in-hospital patient data on procedures, diagnoses and co-morbidities, as well as administrative data (such as length of stay and reimbursement) to the ‘Institute for the Hospital Remuneration System’ (InEK). Patient data are coded in accordance with the German Modification of the ‘International Statistical Classification of Diseases and Related Health Problems 10th Revision’ (ICD-10-GM) and the German ‘Operation and Procedure Classification System’ (OPS). Strict recommendations and regular adjustments by the German Institute for Medical Documentation and Information (Deutsches Institut für Medizinische Dokumentation und Information, DIMDI; Cologne, Germany; https://www.dimdi.de) ensure uniform documentation.

Based on SAS codes (SAS 9.2: SAS Institute Inc., Cary, NC, USA) provided to the Research Data Center of the Federal Bureau of Statistics (Statistisches Bundesamt, DESTATIS; https://www.destatis.de), an excerpt is generated from the above-mentioned dataset and transformed into Stata format (StataCorp, College Station, Texas, USA). Finally, summary tables are retrieved, and statistical tests are conducted on the basis of pre-specified routines supplied to DESTATIS. Results of groups with *n* < 3 are blanked out by DESTATIS for reasons of anonymity, not included in summary statistics and marked as ‘n/a’ in the tables.

### Treatment groups

In-hospital data of all MV procedures performed across Germany between 2009 and 2015 were evaluated. OPS codes were used to identify all admissions relevant for the analysis: (1) percutaneous (OPS codes: 5-35a.x in 2009, 5-35a.4 in 2010, and 5-35a.41 from 2011) and (2) surgical MV reconstruction, including annuloplasty (OPS code: 5-353.1), leaflet repair (OPS code: 5-353.2) and chordae/papillary muscle reconstruction (OPS code: 5-354.12), as well as MV thrombectomy (OPS code: 5-354.11). Cases with MV reconstructions not coded with the above-mentioned OPS codes, or combined surgical mitral, and/or aortic, and/or tricuspid valve procedures, transcatheter aortic valve procedures, or concomitant surgical or percutaneous coronary intervention, as well as cases without documented MV regurgitation (ICD-10 I051, I058, I034 or I241) as main or secondary diagnosis, were excluded from the analysis.

### Baseline characteristics

A number of relevant baseline patient characteristics were evaluated to describe the underlying diseases’ severity and risk-factor composition. The ICD codes used have been discussed in greater detail previously [12]. Based on the European System for Cardiac Operative Risk Evaluation (EuroSCORE) [13], a ‘best-case scenario’ risk score was estimated. For this, besides age and sex, the ICD codes for chronic pulmonary disease (J43*, J44*), for neurological dysfunction (I69*, G81*, R48*), previous cardiac surgery (Z95.1-Z95.4), serum creatinine > 200 µmol/L (N18.0, N18.84; since 201018.4, N18.5), active endocarditis (I33*), unstable angina (I20.0), recent myocardial infarction (I25.20), and pulmonary hypertension (I27*) were utilized. Due to the lack of ‘preoperative state’ and ‘left ventricular function’ data, an inconspicuous state (i.e. no emergency, preserved left ventricular function)—the ‘best-case’—was assumed. In order to allow a direct comparison of the baseline risk factor composition between patients undergoing percutaneous or surgical MV reconstruction, we calculated logistic EuroSCORE values assuming isolated MV procedures for both groups.

### In-hospital outcomes

Clinical in-hospital outcomes include in-hospital mortality and in-hospital complications such as (1) severe bleeding, defined as requiring more than five units of red blood cells (RBC) during the hospital stay (OPS codes: 8-800.7* et seqq., since 2010 8-800.c et seqq), (2) acute kidney injury (ICD code: 17.*), and (3) stroke (ICD codes: I63* and I64).

### Statistical analysis

Differences in baseline characteristics, clinical and economic outcomes between groups were calculated using the Student’s *t* test and the chi-square test for continuous and categorical variables, respectively. In order to identify treatment effects in patients at intermediate risk, a subgroup of patients from 2015 (estimated logistic EuroSCORE ≥ 4% and ≤ 9%, *n* = 2103, [14]) was analyzed with respect to the endpoint in-hospital mortality. Since patients were not randomized to the two treatment options (percutaneous or surgical MV reconstruction), a logistic regression model was used with all available patient and procedural characteristics (as defined by Reinöhl et al. [12]) included as potential confounders (all covariates are listed in Table S1 in the Supplementary Appendix). To account for the correlation of error terms of patients treated in the same hospital, a random intercept was included at the center level. If the respective outcome within this subgroup rarely occurs, however, there may not be enough data with which to model the relationship between the outcome and all potential confounders [15]. Therefore, an additional propensity approach was applied within the same subgroups of patients. First, a logistic regression model was performed on the same patient and procedural characteristics to calculate the propensity score for each patient within the subgroup. The propensity score represents the likelihood that the patient was in the MitraClip arm. Please note that the outcome variables were not used in this step. Then, propensity score adjustment was applied using the propensity score as single continuous covariate [16]. Again, logistic regression models with a random intercept at the center level were conducted. All analyses were carried out using Stata 14 (Stata Corp, College Station, Texas).

## Results

### Patient characteristics

We obtained data on 35,489 admissions to German hospitals for isolated surgical (*n* = 22,825, 64.3%) or percutaneous (*n* = 12,664, 35.7%) MV reconstruction procedures between 2009 and 2015 (Table 1). As expected, baseline patient characteristics differed significantly between groups (*P* < 0.001 for all comparisons; Table 2). Overall, patients undergoing percutaneous MV reconstruction were older, suffered from advanced heart failure symptoms, and showed a higher prevalence of comorbidities compared to patients undergoing surgical MV reconstruction.

**Table 1:** Numbers of percutaneous and surgical MV reconstructions, by year

Procedure	2009	2010	2011	2012	2013	2014	2015	Total
Percutaneous MV repair—no. (%)^a^	108 (3.6)	174 (5.4)	707 (18.4)	1,679 (34.1)	2513 (42.9)	3404 (49.0)	4079 (53.1)	12,664 (35.7)
Surgical MV repair—no. (%)	2923 (96.4)	3030 (94.6)	3139 (81.6)	3250 (65.9)	3343 (57.1)	3537 (51.0)	3603 (46.9)	22,825 (64.3)
All procedures—no.	3031	3204	3846	4929	5856	6941	7682	35,489

Numbers represent procedures, not individual patients; some patients may have undergone more than one procedure. *MV* denotes mitral valve, *no* number

^a^By use of the MitraClip system (Abbott Vascular, Santa Clara, California, USA)

**Table 2:** Baseline characteristics of patients undergoing percutaneous or surgical MV reconstruction

	Percutaneous MV repair (*n* = 12,664)	Surgical MV repair (*n* = 22,825)
Female sex—*%*	40.0	36.2
Age—years	75.6 ± 8.8	61.6 ± 13.4
Estimated logistic EuroSCORE—*%*^a^	13.2 ± 11.1	4.7 ± 5.3
MV regurgitation—*%*	99.5	89.8
Combined MV diseases—*%*^b^	0.05	0.19
Heart failure—*%*		
NYHA II	8.7	11.3
NYHA III or IV	61.5	26.8
Hypertension—*%*	58.2	55.8
CAD—*%*	51.8	11.8
Previous myocardial infarction—*%*		
≤ 4 months earlier	1.8	0.5
≤ 12 months earlier	1.6	0.4
> 12 months earlier	9.2	1.4
Previous CABG—*%*	17.6	2.4
Previous cardiac surgery—*%*	23.6	24.2
Peripheral vascular disease—*%*	7.1	2.0
Carotid disease—*%*	1.9	1.2
COPD—*%*	14.8	7.2
Pulmonary hypertension—*%*	30.7	20.9
Renal disease—*%*		
GFR < 15 ml/min/1.73m^2^	2.6	0.8
GFR < 30 ml/min/1.73m^2^	8.5	1.2
Atrial fibrillation—*%*	63.0	45.2
Diabetes mellitus—*%*	30.7	10.2

*P* < 0.001 for all comparisons. Plus–minus values are means ± standard deviation. Numbers represent procedures, not individual patients; some patients may have undergone more than one procedure. For all variables, *P* < 0.001 for the comparison between percutaneous and surgical MV reconstruction

*CABG* denotes coronary-artery bypass grafting, *CAD* coronary artery disease, *COPD* chronic obstructive pulmonary disease, *GFR* glomerular filtration rate, *MV* mitral valve, and *NYHA* New York Heart Association

^a^The logistic EuroSCORE (European System for Cardiac Operative Risk Evaluation) is calculated by means of a logistic-regression equation; scores range from 0 to 100%, with higher scores indicating greater risk and a score of more than 20% indicating high surgical risk. For calculation of the EuroSCORE, we were able to populate all fields except critical preoperative state and left ventricular function, for which we assumed a low-risk state (i.e., no critical preoperative state and no left ventricular dysfunction) and thus calculated a best-case scenario

^b^This characteristic is the combination of MV stenosis and MV regurgitation

### Numbers of procedures

Since the percutaneous MitraClip approach obtained Conformité Européenne (CE) mark approval in 2008, annual procedural numbers have increased rapidly, from 108 in 2009 to 4079 in 2015. In the same period, surgical MV reconstruction numbers also increased continuously, however, at a more modest rate, from 2923 in 2009 to 3603 procedures in 2015 (see Table 1). Thus, overall procedural numbers more than doubled within 7 years. Among the different surgical MV reconstruction strategies, the combination of annuloplasty and leaflet repair was most frequent throughout the observation period, followed by isolated annuloplasty (Table S2 in the Supplementary Appendix). Annuloplasty was an integral part of almost all (94%) surgical procedures.

**Fig. 1:**
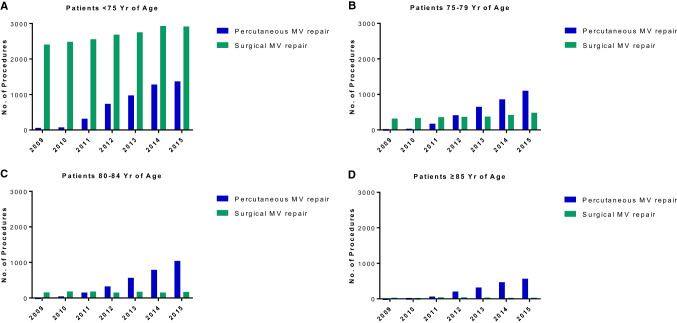
Number of percutaneous and surgical mitral valve reconstruction procedures performed, by age group, 2009–2015. Numbers represent procedures, not individual patients; some patients may have undergone more than one procedure. *MV* denotes mitral valve, *No.* number, *Yr* years

When procedure numbers were examined by age group, the majority (82%) of surgical MV reconstructions were performed in patients younger than 75 years, whereas only 1% of surgical procedures were performed in patients aged ≥ 85 years (Fig. 1). In contrast, 23% of MitraClip procedures were performed in patients aged ≥ 85 years. While the number of percutaneous MV reconstructions increased throughout the observation period in all age groups, there were only minor changes in the numbers of surgical MV replacements among patients 80 years of age or older.

### In-hospital mortality and risk profiles

Although patients with percutaneous edge-to-edge MV reconstruction were at higher operative risk (mean estimated logistic EuroSCORE 13.2% vs 4.7%, *P* < 0.001), overall in-hospital mortality did not statistically differ between groups (2.9% vs 2.8%; *P* = 0.395). This was also true for the subgroup of patients from 2015 (*n* = 2103) who were judged as intermediate operative risk as defined by an estimated logistic EuroSCORE ≥ 4% and ≤ 9% (OR 0.67, 95% CI 0.36–1.25, *P* = 0.21 for multivariable adjustment and OR 0.63, 95% CI 0.34–1.17, *P* = 0.14 for propensity score adjustment, respectively).

**Fig. 2:**
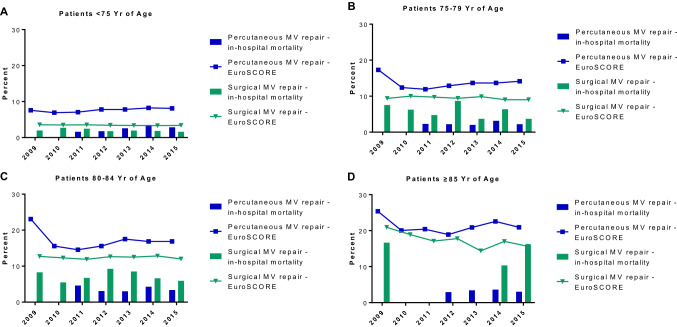
In-Hospital Mortality and Estimated Logistic EuroSCORE among Patients Undergoing Surgical or Percutaneous MV Reconstruction. For calculation of the logistic EuroSCORE (European System for Cardiac Operative Risk Evaluation), we were able to populate all fields except for critical preoperative state and left ventricular function, for which we assumed a low-risk state (i.e., no critical preoperative state and no left ventricular dysfunction) and thus calculated a best-case scenario. Numbers represent procedures, not individual patients; some patients may have undergone more than one procedure. Groups with fewer than three procedures were excluded for reasons of anonymity. *MV* denotes mitral valve, *Yr* years

There were reductions in mortality rates in both treatment groups between 2009 and 2015 (Table S3 in the Supplementary Appendix), but these improvements were smaller for surgical MV repair (from 3.1 in 2009 to 2.8% in 2015) than for percutaneous MV procedures (from 6.5 to 2.9%). However, starting with 2012, both risk estimate and in-hospital mortality increased again in the percutaneous edge-to-edge group. In 2015, in-hospital mortality with percutaneous MV repair was higher than with surgical MR repair (2.8% vs 2.2%, *P* = 0.081). This was mainly driven by an increase in in-hospital mortality in patients aged younger than 75 years in the percutaneous edge-to-edge group (1.6% in 2011 vs 2.8% in 2015). In contrast, in-hospital mortality with percutaneous MV repair was lower than with surgical MR repair in all other age groups, despite higher operative risk as estimated by the logistic EuroSCORE (Fig. 2).

### Complications

Overall, the frequencies of documented in-hospital complications differed markedly between the two procedures. Bleeding was the most frequently reported complication, occurring significantly more often in surgery (12.1% vs 2.0%, *P* < 0.001). Moreover, surgical patients more often suffered a stroke compared to patients undergoing a percutaneous edge-to-edge approach (1.8% vs 0.7%, *P* < 0.001). Acute kidney injury occurred, however, more frequently in the percutaneous group (7.3% vs 4.0%, *P* < 0.001).

## Discussion

We report nationwide and contemporary data on in-hospital outcomes for isolated percutaneous (12,664 procedures) and surgical MV reconstructions (22,825 procedures) in Germany between 2009 and 2015. To the best of our knowledge, the present study represents the largest dataset and the sole nationwide analysis on this topic in the literature [17–24].

During the observation period, annual overall procedural numbers more than doubled, with a substantial increase in percutaneous edge-to-edge use in all age-groups. However, in contrast to the latest experience with the introduction of transcatheter aortic valve replacement in Germany [12], the increase in percutaneous MV procedures did not occur at the expense of surgical numbers. This indicates that the availability of the percutaneous edge-to-edge approach has led to the treatment of a previously untreated population that may have previously been considered too sick for surgery.

Our data show improvemed in-hospital outcomes over time for both percutaneous and surgical MV repair. For the surgical reconstruction this is probably due to changes and developments in technical strategies over the years—for the group undergoing percutaneous edge-to-edge repair it might be a combination of factors such as a “learning curve” effect on procedural skills and improvements in patient selection and care. Furthermore, a shift of high-risk patients to the percutaneous edge-to-edge approach may have contributed to both the positive development for surgical reconstruction and to the moderate renewed rise in in-hospital mortality in the percutaneous edge-to-edge group observed since 2013.

While surgical MV procedures were predominantly performed in patients younger than 75 years, the vast majority of patients aged 75 years and older underwent percutaneous edge-to-edge MV repair. The present study shows a favorable safety profile of the percutaneous edge-to-edge approach especially in older patients, with low in-hospital mortality and complication rates. This is of particular note given the adverse risk-profile of the patients treated.

Our data confirm the results from the small randomized-controlled Endovascular Valve Edge-to-Edge Repair Study (EVEREST II) [9] as well as those of an early review of 12 prospective observational studies [24] that identified the percutaneous MV repair as a safe and feasible option for high-risk patients. In-hospital mortality and stroke rates in other published registries are similar to our data (in-hospital mortality: 2.0–3.0%, stroke: 0.2–1.0%) [17–23]. However, the mean logistic EuroSCORE estimates in the percutaneous MV group were lower in our study (13.2% compared to 20.0–23.0%). This may be due to differences in data-collection methods and definitions (prospective registries vs. retrospective administrative data, assumed best-case scenario), but it may also point to systematic differences in the populations covered. Yet, even the largest registry reported only a small fraction of procedural numbers compared to the dataset analyzed in this study (828 vs 12,644 patients) [20, 25, 26]. Moreover, the present study is the first that analyzes outcome data of percutaneous and surgical MV repair in patients who are judged at intermediate operative risk. Our data suggest similar in-hospital mortality with both treatments in this patient cohort. Prospective trials are warranted to clarify whether percutaneous edge-to-edge MV repair may also lead to favorable long-term outcomes in intermediate-risk patients.

## Limitations

As our data are observational, no causal inference can be concluded. Besides, already the substantially different cohorts would preclude any direct comparison of effectiveness. Therefore the present analysis did not attempt to do so but rather concentrates on the description of cohorts and in-hospital outcome and their evolution over time. To this end, we would consider isolated MV repair as the most appropriate comparator. While it is certainly right that e.g. concomitant tricuspid surgery would be guideline conform, it might potentially add theatre and/or bypass-time and complication rates thus dysbalancing the cohorts.

Other than that, our dataset has several specific limitations: First, it is based on administrative data reporting diagnoses and procedures for reimbursement purposes. Coding errors are inevitable and, therefore, the safety profile and complication rates may be incompletely depicted. Their influence, however, may be acceptable for the following reasons: (1) about 20% of all cardiovascular DRGs are checked by independent physician task forces from health insurers, which should ensure a high level of data reliability and quality; (2) they would be evenly spread between both cohorts; (3) as they potentially upgrade DRG-reimbursement without being linked to quality assurance reportings, overreporting may be more likely than underreporting. Still, we would be unable to discern if, e.g. the association of edge-to-edge repair with increased incidence of kidney injury was due to the influence of age/co-morbidities, due to medical reasons, or due to coding.

Lack of coding might also impact on the definition of aetiology of MV regurgitation. Currently, the influence of the nature/proportionality of MR [27] and the best way to measure it [28, 29] is under debate. It is conceivable that some of the patients had MV regurgitation due to past endocarditis rather than secondary MV disease proper. Given that aetiology coding would not be mandatory for past endocarditis and that—on the other hand—active endocarditis might not receive surgical MV reconstruction as a primary intention, we cannot exclude this potential confounder in our dataset. Second, the administrative dataset lacks relevant clinical information such as echocardiographic findings or anatomical characteristics. Therefore, only an approximation of the logistic EuroSCORE—in fact, a ‘best-case scenario’ estimate—was used. However, the size and nature of the data set have strengths in terms of completeness and masking of data from clinicians. Third, commonly used endpoints such as improvement of MV regurgitation, symptoms relief, or quality of life improvements were not reported, which is a common limitation of real-world data. In fact, administrative databases or registries are powerful tools to characterize the safety profile of a new drug or device, while randomized controlled trials are better suited to assess efficacy. In this context, COAPT [11] showed a clear benefit compared to medical therapy even though the issue of proportionality [27] may contribute to this interpretation. Here, the results of MATTERHORN (NCT02371512) will allow a more complete picture. Fourth, long-term follow up data are missing, as DESTATIS provides no longitudinal data or cross-links with other clinical or administrative datasets. Finally, the present analysis relies on data from the German healthcare system and other countries’ experiences may differ. Fifth, we are aware that the logistic EuroSCORE may not be the currently most widely suggested method for risk stratification. The structure of the DESTATIS dataset, however, precludes calculation of the STS- or EuroSCORE 2 with our dataset. Following suggestions for conversion of scores [14], we considered a logistic EuroSCORE of 9 equivalent to a STS score of 4 and, therefore, the threshold for intermediate and high pre-operative risk.

## Conclusion

In conclusion, we analyzed all isolated surgical and percutaneous MV reconstructions performed in Germany between 2009 and 2015. Annual overall procedural numbers more than doubled in this period, with a massive increase in the percutaneous edge-to-edge technique and a slight increase in surgical MV reconstructions. Patients undergoing the percutaneous approach tended to be older and at higher procedural risk than those undergoing surgical MV repair, indicating that the availability of a percutaneous strategy has enabled the treatment of a previously untreated population. In hospital-mortality improved over time for both percutaneous and surgical MV repair. Our large-scale dataset shows a favorable safety profile of the percutaneous edge-to-edge approach in both patients at high and intermediate operative risk.

## Electronic supplementary material

Below is the link to the electronic supplementary material.

Supplementary file1 (DOCX 22 kb)

## Data Availability

The data used are stored following data protection guidelines in an enclosed virtual space of the Federal Bureau of Statistics to which data requests must be addressed.
