# Supplementary material for: Impact of the introduction of percutaneous edge-to-edge mitral valve reconstruction on clinical practice in Germany compared to surgical valve repair
Source: Clin Res Cardiol. 2020 May 27;110(5):620–7. doi: 10.1007/s00392-020-01675-0 (PMC8099833; doi:10.1007/s00392-020-01675-0)
Supplement: Supplementary file 1 — Supplementary file1 (DOCX 22 kb) [file 392_2020_1675_MOESM1_ESM.docx]

**Supplementary Appendix**

Impact of the introduction of percutaneous edge-to-edge mitral valve reconstruction on clinical practice in Germany

| **Table of Contents** | **Page** |
| --- | --- |
| Acknowledgments | 2 |
| Table S1: Covariates included in the multivariable logistic regression model analyzing in-hospital mortality in the subgroup of patients at intermediate operative risk undergoing percutaneous or surgical MV repair in 2015 | 3 |
| Table S2: Numbers of surgical MV reconstructions, according to year | 4 |
| Table S3: In-hospital mortality in percutaneous and surgical MV reconstructions, according to year | 5 |

**Acknowledgements:**

We are indebted to the excellent assistance of Dipl.-Stat. Angelika Gerlach of the Study Centre of the Medical Center – University of Freiburg and coder Marion Rüdiger for their support in the acquisition of the data. We would also like to thank the staff of the Research Centers of the Federal Bureau of Statistics and the statistical offices of the federal states for providing the data and the technical support during the analysis. Everyone who contributed significantly to this work has been listed.

**Table S1:** Covariates included in the multivariable logistic regression model analyzing in-hospital mortality in the subgroup of patients at intermediate operative risk undergoing percutaneous or surgical MV repair in 2015*

| **Covariates** |
| --- |
| Female sex |
| Age |
| Estimated logistic EuroSCORE † |
| MV regurgitation (vs. combined MV diseases ‡) |
| NYHA II |
| NYHA III or IV |
| Hypertension |
| CAD |
| Previous myocardial infarction |
| ≤4 months earlier |
| ≤12 months earlier |
| >12 months earlier |
| Previous CABG |
| Previous cardiac surgery |
| Peripheral vascular disease |
| Carotid disease |
| COPD |
| Pulmonary hypertension |
| Renal disease |
| GFR <15 ml/min/1.73m² |
| GFR <30 ml/min/1.73m² |
| Atrial fibrillation |
| Diabetes mellitus |
| * Patients at intermediate risk undergoing MV therapy in 2015 (n = 2,103) were identified by an estimated logistic EuroSCORE ≥4% and ≤9%. *CAB* denotes coronary-artery bypass grafting, *CAD* coronary artery disease, *COPD* chronic obstructive pulmonary disease, *GFR* glomerular filtration rate, *MV* mitral valve, and *NYHA* New York Heart Association.  † The logistic EuroSCORE (European System for Cardiac Operative Risk Evaluation) is calculated by means of a logistic-regression equation; scores range from 0 to 100%, with higher scores indicating greater risk and a score of more than 20% indicating high surgical risk. For calculation of the EuroSCORE, we were able to populate all fields except critical preoperative  state and left ventricular function, for which we assumed a low-risk state (i.e., no critical preoperative state and no left ventricular dysfunction) and thus calculated a best-case scenario.  ‡ This characteristic is the combination of MV stenosis and MV regurgitation. |

**Table S2:** Numbers of surgical MV reconstructions, according to year*

| **Procedure** | **2009** | **2010** | **2011** | **2012** | **2013** | **2014** | **2015** | **Total** |
| --- | --- | --- | --- | --- | --- | --- | --- | --- |
| Annuloplasty only – *no.* | 680 | 641 | 597 | 624 | 630 | 603 | 646 | 4,421 |
| Leaflet repair only – *no.* | 130 | 117 | 115 | 120 | 142 | 134 | 88 | 758 |
| Thrombectomy only – *no.* | 14 | 9 | 4 | 9 | 10 | 19 | 16 | 65 |
| Chordae/papillary muscle reconstruction only – *no.* | 62 | 56 | 58 | 38 | 65 | 41 | 49 | 320 |
| Combined annuloplasty and leaflet repair – *no.* | 1,127 | 1,219 | 1,264 | 1,282 | 1,167 | 1,228 | 1,363 | 7,287 |
| Combined annuloplasty and thrombectomy – *no.* | 0 | NA | 0 | NA | NA | 0 | 4 | 4 |
| Combined annuloplasty and chordae/papillary muscle reconstruction – *no.* | 405 | 406 | 472 | 491 | 611 | 644 | 624 | 3,029 |
| Combined leaflet repair and thrombectomy – *no.* | NA | NA | 0 | NA | NA | NA | 9 | 9 |
| Combined leaflet repair and chordae/papillary muscle reconstruction – *no.* | 18 | 20 | 19 | 19 | 16 | 27 | 33 | 119 |
| Combined thrombectomy and chordae/papillary muscle reconstruction – *no.* | 0 | NA | 0 | NA | 0 | NA | 3 | 3 |
| Combined annuloplasty, leaflet repair and chordae/papillary muscle reconstruction – *no.* | 483 | 555 | 610 | 661 | 694 | 835 | 782 | 3,838 |
| Other combinations – *no.* |  |  |  |  |  |  |  | 2,972 |
| * Numbers represent procedures, not individual patients; some patients may have undergone more than one procedure. *MV* denotes mitral valve, *no.* number, *NA* not available. | | | | | | | | |

**Table S3:** In-hospital mortality in percutaneous and surgical MV reconstructions, according to year*

|  |  | **2009** | **2010** | **2011** | **2012** | **2013** | **2014** | **2015** | **Total** | **Trend (P value)** |
| --- | --- | --- | --- | --- | --- | --- | --- | --- | --- | --- |
| **Percutaneous MV repair †** | No. | 108 | 174 | 707 | 1,679 | 2,513 | 3,404 | 4,079 | 12,664 |  |
|  | Age, *years* ‡ | 73.0 | 75.2 | 74.5 | 74.8 | 75.5 | 75.8 | 76.2 | 75.6 |  |
|  | Estimated logistic EuroSCORE § | 13.74% | 11.94% | 11.08% | 11.92% | 13.19% | 13.60% | 13.75% | 13.18% |  |
|  | In-hospital mortality | 6.48% | 3.45% | 2.26% | 2.26% | 2.63% | 3.61% | 2.82% | 2.93% |  |
| **Surgical MV repair** | No. | 2,923 | 3,030 | 3,139 | 3,250 | 3,343 | 3,537 | 3,603 | 22,825 |  |
|  | Age, *years* ‡ | 61.6 | 61.6 | 62.2 | 61.5 | 61.5 | 61.0 | 61.9 | 61.6 |  |
|  | Estimated logistic EuroSCORE § | 4.86% | 4.86% | 4.92% | 4.72% | 4.69% | 4.53% | 4.65% | 4.74% |  |
|  | In-hospital mortality | 3.11% | 3.27% | 2.96% | 2.92% | 2.45% | 2.66% | 2.19% | 2.77% |  |
| * Numbers represent procedures, not individual patients; some patients may have undergone more than one procedure. *MV* denotes mitral valve, *No.* number.  † By use of the MitraClip system (Abbott Vascular, Santa Clara, California, USA)  ‡ Values show mean age.  § For calculation of the logistic EuroSCORE, we were able to populate all fields except critical preoperative state and left ventricular function, for which we assumed a low-risk state (i.e., no critical preoperative state and no left ventricular dysfunction) and thus calculated a best-case scenario. | | | | | | | | | | |
